# Thermal Properties of the Mixed *n*-Octadecane/Cu Nanoparticle Nanofluids during Phase Transition: A Molecular Dynamics Study

**DOI:** 10.3390/ma10010038

**Published:** 2017-01-05

**Authors:** Qibin Li, Yinsheng Yu, Yilun Liu, Chao Liu, Liyang Lin

**Affiliations:** 1 Chongqing Key Laboratory of Heterogeneous Material Mechanics, College of Aerospace Engineering, Chongqing University, Chongqing 400044, China; jack_linliyang@cqu.edu.cn; 2 Key Laboratory of Low-grade Energy Utilization Technology & System, Ministry of Education, College of Power Engineering, Chongqing University, Chongqing 400044, China; 20123858@cqu.edu.cn (Y.Y.); liuchao@cqu.edu.cn (C.L.); 3 State Key Laboratory for Strength and Vibration of Mechanical Structures, School of Aerospace Engineering, Xi’an Jiaotong University, Xi’an 710049, China

**Keywords:** *n*-octadecane, Cu nanoparticle, phase transition, thermal conductivity, molecular dynamics simulation

## Abstract

Paraffin based nanofluids are widely used as thermal energy storage materials and hold many applications in the energy industry. In this work, equilibrium and nonequilibrium molecular dynamics simulations are employed to study the thermal properties of the mixed nanofluids of *n*-octadecane and Cu nanoparticles during phase transition. Four different nanofluids systems with different mass ratios between the *n*-octadecane and Cu nanoparticles have been studied and the results show that Cu nanoparticles can improve the thermal properties of *n*-octadecane. The melting point, heat capacity and thermal conductivity of the mixed systems are decreased with the increasing of the mass ratio of *n*-octadecane.

## 1. Introduction

Today, severe challenges are encountered during the development of modern society in the whole world, such as energy crisis, environmental pollution, global climate warming, etc. Use of renewable and clean energy and improving the energy efficiency are the main solutions for the above challenges. One of the most attractive approaches is the thermal energy storage in phase change materials (PCMs) [1], which can not only provide sufficient renewable energy, but also can optimize the energy utilization. Actually, the PCMs have many potential applications in a lot of fields, such as building energy saving, thermal insulation of woven textile fabrics, electronic component cooling, solar thermal power generation, waste heat recovery, and so on [2].

Paraffin is one of the typical PCMs for its large latent heat, low cost, chemical stability and non-toxicity. However, the thermal conductivity of paraffin is low, which limits its application in thermal energy storage. However, the thermophysical properties of working fluid can be significantly improved by adding nanoparticles into it, which is the so-called nanofluid/nanocomposite [3]. Therefore, paraffin based nanofluid/nanocomposite have been extensively studied since the last decade. Zhu et al. [4,5] experimentally studied the thermal properties of paraffin based PCMs by adding Cu, Al, carbon nanotube and graphite nanoparticles. The thermal conductivities of PCMs with Cu and graphite were improved significantly. Besides, the thermal properties of PCMs with nanoparticles are stable after a long-time service of thermal cycling. Ho and Gao [6] prepared Al_2_O_3_/paraffin emulsion and found that both the thermal conductivity and dynamic viscosity of the emulsion nonlinearly increase with the mass fraction of the nanoparticles. However, up to now, a fundamental understanding of the enhancing mechanism of the thermal properties for the paraffin based nanofluids/nanocomposite is still lacking. The systematical studies of the interactions between nanoparticles and paraffin are helpful to the understanding of the enhancing mechanism and the design of high performance PCMs.

Due to the nanoscale dimension of nanoparticles, it is difficult to investigate the interactions between nanoparticles and paraffin by conventional experimental methods. Therefore, several alternative approaches are proposed to investigate the thermal properties of paraffin based nanofluid/nanocomposite, among which molecular dynamics (MD) simulation [7] has been proved to be a powerful tool in studying the nanoscale thermal and mechanical behaviors [8,9]. Rao et al. [10,11] used the MD simulation to study the thermal properties of a nano-capsule, that is the *n*-octadecane, *n*-nonadecane, *n*-eicosane, *n*-heneicosane or *n*-docosane as a core and SiO_2_ as a shell. Their results agreed well with experiments and verified the validity of studying the thermal properties of the nanofliud/nanocomposite PCMs via MD simulations from the nanoscale. Wang et al. [12] also studied the octadecane–water PCM via MD simulations. They found that the heat capacity of octadecane slurry decreased with the mass ratio of the octadecane. Since the nanofluid/nanocomposite PCMs usually consist of complicated and various components, MD simulation is an ideal method to investigate the thermal properties of the nanofluid/nanocomposite PCMs. As a common additive of nanofluids, Cu nanoparticles can improve the thermal properties of working fluid [4,13]. Therefore, in this work, MD simulations are employed to investigate the thermal properties of *n*-octadecane/Cu nanoparticle nanofluids during phase transition.

## 2. Model and Computational Method

In MD simulation, the motions of the atoms are governed by Newton’s second law, while the force applied on every atom is determined by positions of the atoms and the corresponding molecular force fields.

### 2.1. Simulation Model and Molecular Force Fields

**Figure 1:**
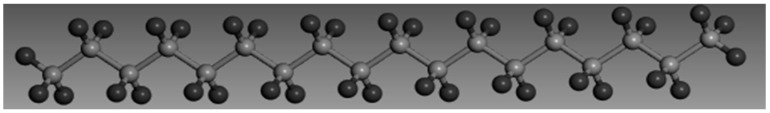
Molecular structure of *n*-octadecane. (Grey spheres: Carbon atoms, black spheres: Hydrogen atoms.)

**Figure 2:**
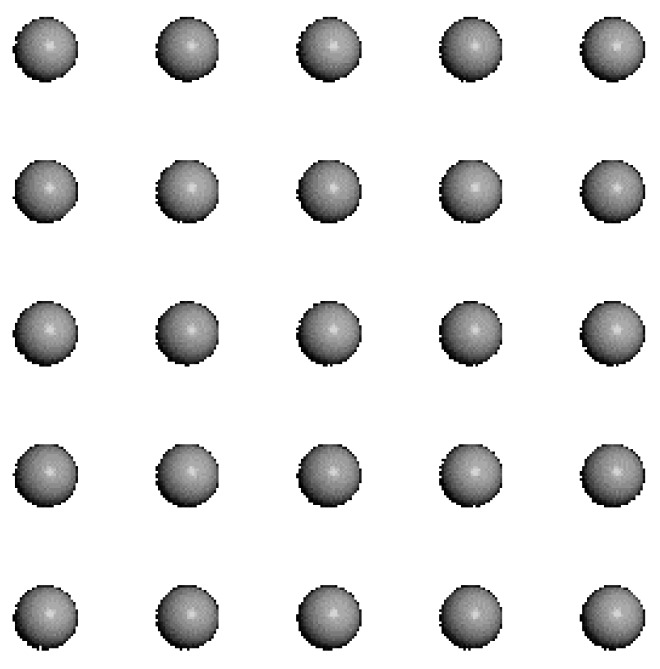
Atoms configuration of Cu nanoparticles.

In general, *n*-octadecane is the common ingredient in paraffin. The molecular structure of *n*-octadecane is a straight chain consisting of 18-alkanes (CH_3_–(CH_2_)_16_–CH_3_), as shown in Figure 1. The Cu nanoparticle studied in this work consists of 2 × 2 × 2 unit cell (64 Cu atoms) as shown in Figure 2. By considering the complicated interaction of alkane, the condensed-phase optimized molecular potentials for atomistic simulation studies (COMPASS) force field [14] is used to describe the interactions of the investigated 18-alkanes and Cu atoms.

**Figure 3:**
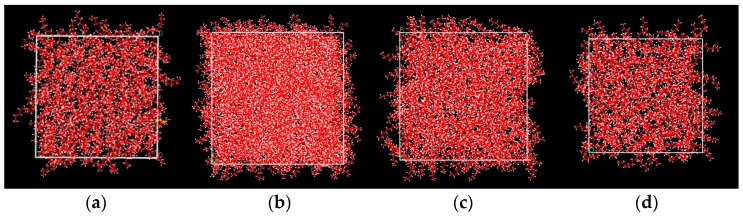
Initial configuration of systems. (**a**) 128 *n*-octadecane molecules; (**b**) 1 Cu nanoparticle and 512 *n*-octadecane molecules; (**c**) 1 Cu nanoparticle and 256 *n*-octadecane molecules; (**d**) 1 Cu nanoparticle and 128 *n*-octadecane molecules.

Four systems are created to study the effect of Cu nanoparticles on the thermal properties of the *n*-octadecane/Cu nanoparticle nanofluids, which are (a) 128 *n*-octadecane molecules (2304 carbon atoms and 4864 hydrogen atoms) in a simulation box of 4.2 × 4.2 × 4.2 nm^3^; (b) 1 Cu nanoparticle and 512 *n*-octadecane molecules (9216 carbon atoms, 19,456 hydrogen atoms and 64 Cu atoms) in a simulation box of 6.5 × 6.5 × 6.5 nm^3^; (c) 1 Cu nanoparticle and 256 *n*-octadecane molecules (4608 carbon atoms, 9728 hydrogen atoms and 64 Cu atoms) in a simulation box of 5.8 × 5.8 × 5.8 nm^3^; and (d) 1 Cu nanoparticle and 128 *n*-octadecane molecules (2304 carbon atoms, 4864 hydrogen atoms and 64 Cu atoms) in a simulation box of 4.7 × 4.7 × 4.7 nm^3^. The initial configurations of the studied systems, as shown in Figure 3, are obtained by the AMORPHOUS CELL module of Materials Studio (Accelrys Software Inc., San Diego, CA, USA) [15], in which we set the initial temperature of the systems at 320 K.

### 2.2. Computational Method

The simulations are performed by Materials Studio. Periodic boundary conditions are applied in X, Y, Z directions. The timestep is set as 1 fs in the simulations. Since, in this work, we focus on the thermal properties of the *n*-octadecane/Cu nanoparticle nanofluids during the phase transition, the four systems are equilibrated at 285 K, 295 K, 300 K, 305 K, 310 K, 320 K, 325 K and 330 K in NVT (canonical) ensemble for 50 ps in the FORCITE module of Materials Studio, respectively, to calculate their thermal properties near melting point. Note that the melting point for pure *n*-octadecane is about 301 K [16]. These simulations are the so-called equilibrium MD (EMD) simulations. Some of the thermal properties are analyzed by our homemade PERL script. The Berendsen method [17] is used to control the temperature of the simulation systems and the Velocity–Verlet algorithm is applied to update the atomic motions.

### 2.3. Thermal Conductivity Calculation

**Figure 4:**
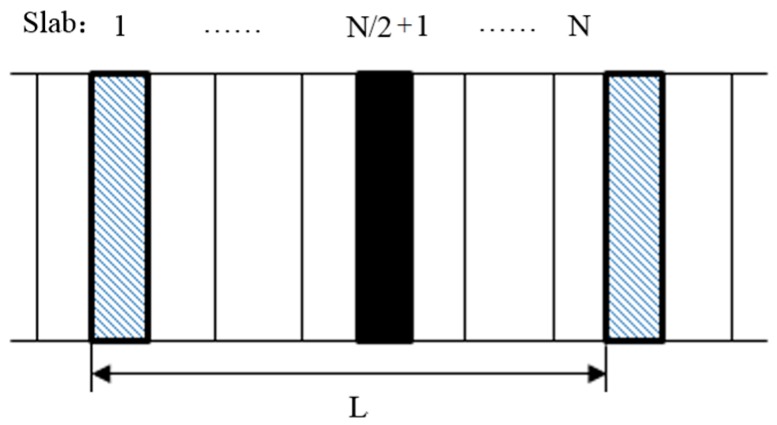
NEMD for calculating thermal conductivity.

Then, the above equilibrium systems are simulated by nonequilibrium MD (NEMD) based on our homemade PERL script to compute their thermal conductivities. The system is divided into 20 slabs along the X direction, as shown in Figure 4. The Muller–Plathe algorithm [18,19] is used to exchange the kinetic energy of particles in the first and eleventh slabs, every 100 steps. The stable temperature gradient is generated after performing the exchange process for 50,000 timesteps. Then, the thermal conductivity of the *n*-octadecane/Cu nanoparticle nanofluids is calculated in the following 100,000 timesteps. Here, the total exchanged kinetic energy during the MD simulation by using the Muller–Plathe method is recorded and output. Therefore, the average heat flux of the *n*-octadecane/Cu nanoparticle nanofluids in the X direction is calculated through dividing the total exchanged kinetic energy by time and the cross section area, i.e., Y dimension × Z dimension of the simulation box. Thus, according to the Fourier law of thermal conduction, the thermal conductivity of the *n*-octadecane/Cu nanoparticle nanofluids can be calculated through dividing the heat flux by the temperature gradient in the X direction. Note that due to the nanoscale dimension of the *n*-octadecane/Cu nanoparticle nanofluids, we ignore the convection heat transfer in this work. The details of this method are described in References [18,19]. The other computational parameters for the NEMD are the same as that in Section 2.2.

## 3. Results and Discussion

Indeed, the thermal properties obtained from MD are usually dependent on the atomic force field used in the simulations. Therefore, the thermal properties of the *n*-octadecane/Cu nanoparticle nanofluids during phase transition discussed in this work are based on the COMPASS force field. The numerical values of the melting point, heat capacity and thermal conductivity of the *n*-octadecane/Cu nanoparticle nanofluids may be different for different atomic force fields. However, we believe that the trends of these thermal properties of the mass ratio of *n*-octadecane are similar.

### 3.1. Diffusion Coefficient

Generally, for a given material, the diffusion coefficient of the liquid state is larger than that of its solid state. Therefore, the phase transition of the *n*-octadecane/Cu nanoparticle nanofluids is calibrated by the transition of the relationship between the diffusion coefficient and temperature. The diffusion coefficient of the system is determined by:

(1) $$D=\frac{1}{6}\underset{t\to \infty }{\lim}\frac{d}{d_{t}}\sum_{i=1}^{N}MSD$$

where the *MSD* is the mean square displacement of atoms in the system [20], *t* is the simulation time. The *MSD* is a typical dynamic parameter defined as:

(2) $$MSD=\langle {\left|\vec{r}\left(t\right)-\vec{r}\left(0\right)\right|}^{2}\rangle$$

$\vec{r}\left(t\right)$ is the position of the atom at the time *t*.

**Figure 5:**
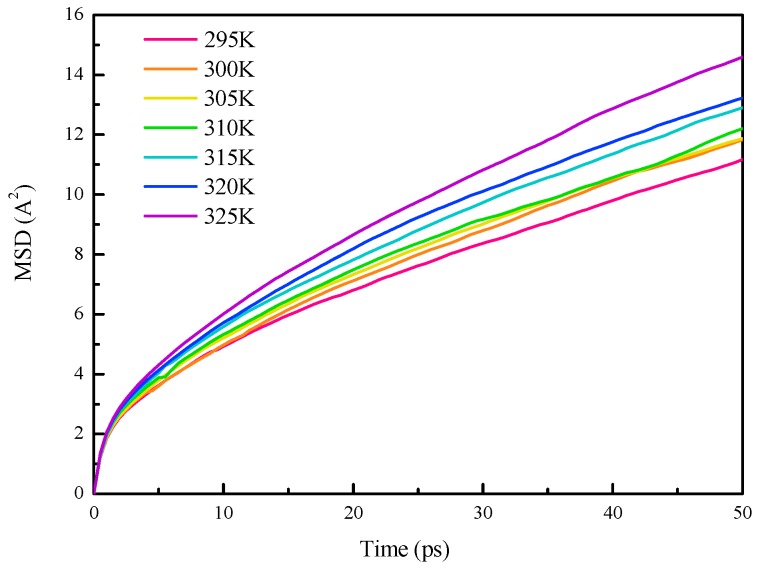
MSD of system (c) (1 Cu nanoparticle and 256 *n*-octadecane molecules) for different system temperatures.

The *MSD* of system (c) (1 Cu nanoparticle and 256 *n*-octadecane molecules) for different temperatures, from 295 K to 325 K during the MD simulations, is plotted in Figure 5. The *MSD* increases with the simulation time and the relationships between the *MSD* and simulation time are almost linear after sufficient simulation time, e.g., 30 ps in our simulations. Thus, the diffusion coefficient is defined as the slope of the *MSD*-simulation time curves for the simulation time larger than 30 ps. Besides, the *MSD* increases as the temperature increases, which is because the atoms have a higher degree of mobility for higher temperature.

**Figure 6:**
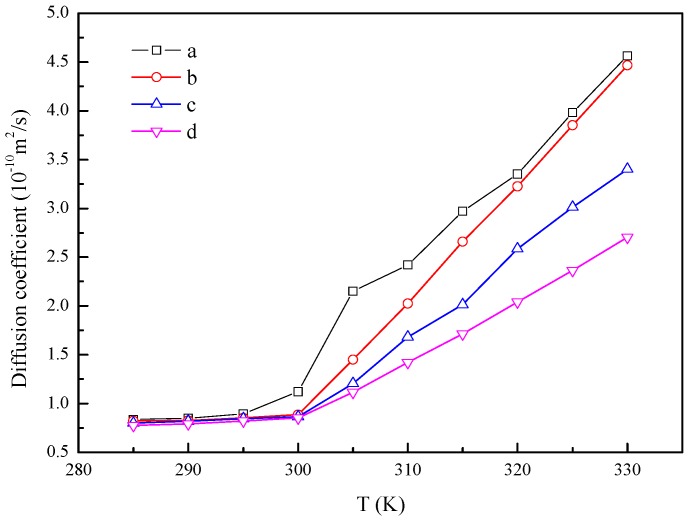
Diffusion coefficient of the different *n*-octadecane/Cu nanoparticle nanofluids with different system temperatures. (**a**) 128 *n*-octadecane molecules; (**b**) 1 Cu nanoparticle and 512 *n*-octadecane molecules; (**c**) 1 Cu nanoparticle and 256 *n*-octadecane molecules; (**d**) 1 Cu nanoparticle and 128 *n*-octadecane molecules.

Next, the diffusion coefficient of the studied systems with different temperatures is calculated and presented in Figure 6. It is shown that the diffusion coefficients of the four systems are almost constant for the system temperature lower than 295 K, which means that the four systems are at the solid state. Then, the transition of the diffusion coefficient–temperature curves for the four systems occurs at about 300 K. The slope of the diffusion coefficient–temperature curves after transition decreases from system (a) to system (d), as shown in Figure 6. Therefore, it can be concluded that the sequence of the phase transition is from system (a) to system (d) as the temperature increases. Furthermore, the melting point of the *n*-octadecane/Cu nanoparticle nanofluids increases as the mass ratio of the Cu nanoparticle increases. Further study has shown that the increasing of the melting point is proportional to the mass ratio of the Cu nanoparticle in *n*-octadecane.

### 3.2. Heat Capacity

**Figure 7:**
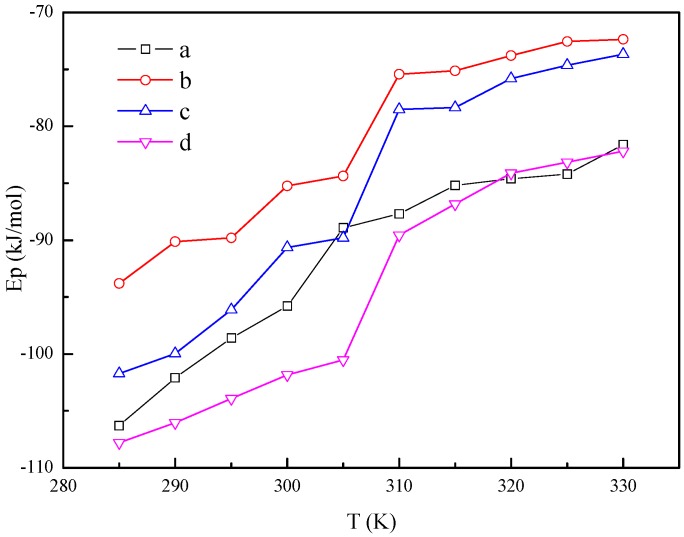
Potential energy of the *n*-octadecane/Cu nanoparticle nanofluids with different temperatures. (**a**) 128 *n*-octadecane molecules; (**b**) 1 Cu nanoparticle and 512 *n*-octadecane molecules; (**c**) 1 Cu nanoparticle and 256 *n*-octadecane molecules; (**d**) 1 Cu nanoparticle and 128 *n*-octadecane molecules.

The potential energy of the systems also increases with the system temperature increasing, as shown in Figure 7. The transitions are also observed in the potential energy–temperature curves near the melting point. Hence, the latent heat of phase transition is partly represented by the variation of the potential energy. Here, the variation of the potential energy near the melting point is 18.9 kJ/mol, 22.3 kJ/mol, 28.2 kJ/mol and 27.4 kJ/mol for system (a), system (b), system (c) and system (d), respectively. So, the *n*-octadecane/Cu nanoparticle nanofluids have larger latent heat of phase transition than that of pure *n*-octadecane, which is beneficial for the thermal energy storage.

**Figure 8:**
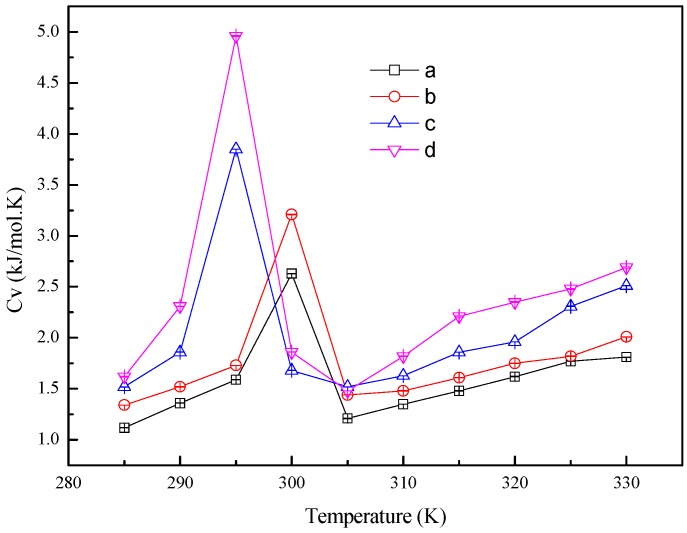
Heat capacity of the *n*-octadecane/Cu nanoparticle nanofluids with different temperatures. (**a**) 128 *n*-octadecane molecules; (**b**) 1 Cu nanoparticle and 512 *n*-octadecane molecules; (**c**) 1 Cu nanoparticle and 256 *n*-octadecane molecules; (**d**) 1 Cu nanoparticle and 128 *n*-octadecane molecules.

Heat capacity (*C_V_*) is an important parameter for the thermodynamic system. In MD simulation, *C_V_* is defined as [10]:

(3) $$C_{V}={\left(\frac{\partial E}{\partial T}\right)}_{v}=\frac{\langle \delta {\left(E_{k}+U+PV\right)}^{2}\rangle }{k_{B}T^{2}}$$

where *E_k_*, *U*, *P*, *V*, *k_B_* and *T* are kinetic energy, potential energy, pressure, volume, Boltzmann constant and temperature, respectively. As shown in Figure 8, the heat capacity has the maximum value near the melting point due to the latent heat of phase transition. The results show that heat capacity of working fluid during phase transition is influenced by the nanoparticle additives. The heat capacity of the *n*-octadecane/Cu nanoparticle nanofluid is large with a high content of the Cu nanoparticle, which is because the Cu nanoparticles can absorb more thermal energy. This result agrees with the aforementioned discussion of the potential energy. The calculated heat capacity of pure *n*-octadecane, system (a), also agrees with that of the octadecane slurry studied in the previous literature [12].

### 3.3. Thermal Conductivity

The thermal conductivity of nanofluid has been extensively studied for many years. First, the thermal conductivity of *n*-octadecane/Cu nanoparticle nanofluids is calculated through a theoretical approach to verify the MD simulation results. In general, the thermal conductivity of nanofluid (*k_eff_*) can be calculated by the relations between the thermal conductivity of nanoparticles (*k_p_*) and the thermal conductivity of working fluid (*k_f_*), which has the following general formula [3]:

(4) $$k_{eff}=\frac{k_{p}\alpha_{p}{\left(dT/dx\right)}_{p}+k_{f}\alpha_{f}{\left(dT/dx\right)}_{f}}{\alpha_{p}{\left(dT/dx\right)}_{p}+\alpha_{f}{\left(dT/dx\right)}_{f}}$$

However, Equation (4), which is limited by many factors, is usually inaccurate to predict the thermal conductivity of nanofluid. Thus, some modified models are proposed, such as the Maxwell model:

(5) $$\frac{k_{eff}}{k_{f}}=\frac{k_{p}+2k_{f}-2\phi \left(k_{f}-k_{p}\right)}{k_{p}+2k_{f}+\phi \left(k_{f}-k_{p}\right)}$$

where *φ* is the volume fraction of nanoparticles. Here, Equation (5) is used to calculate the thermal conductivity of system (d) at 320 K.

The results obtained by NEMD and the Maxwell model are listed in Table 1. Furthermore, the maximum deviation between the present NEMD result and theoretical prediction is about 5.1%, which further verifies the validity of the MD simulation results.

**Figure 9:**
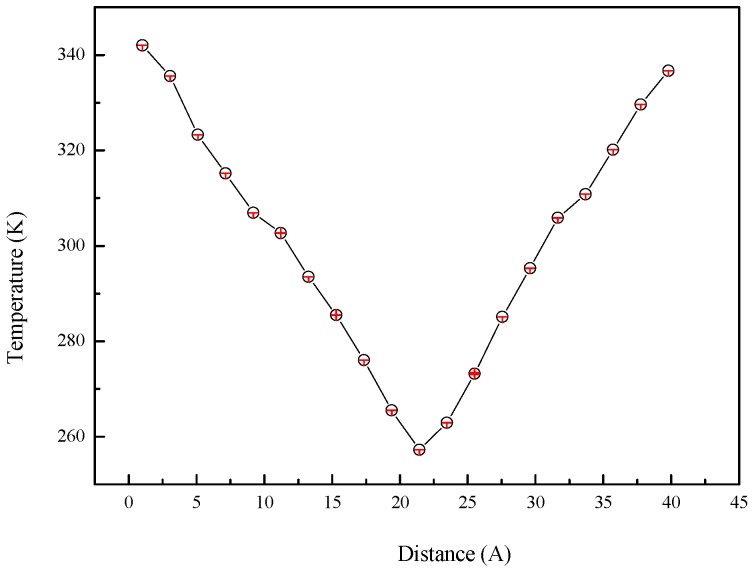
Temperature profile of system (a) (128 *n*-octadecane molecules) in NEMD simulation with an average system temperature of 305 K.

As mentioned in Section 2.3, the thermal conductivity is calculated based on the Fourier law of thermal conduction, so that the temperature gradient is an important parameter in the calculation of thermal conductivity. The temperature of every slab for system (a) is shown in Figure 9. The system temperature linearly decreases from the heat source (first slab) to the cold source (eleventh slab) which guarantees the validity of the Fourier law of thermal conduction. The temperature of the intermediate slab (sixth or sixteenth slab) is about 305 K and the calculated thermal conductivity represents the thermal conductivity of the system at this temperature. The temperature gradient is the temperature difference between the heat source and cold source divided by the distance of the two slabs.

**Figure 10:**
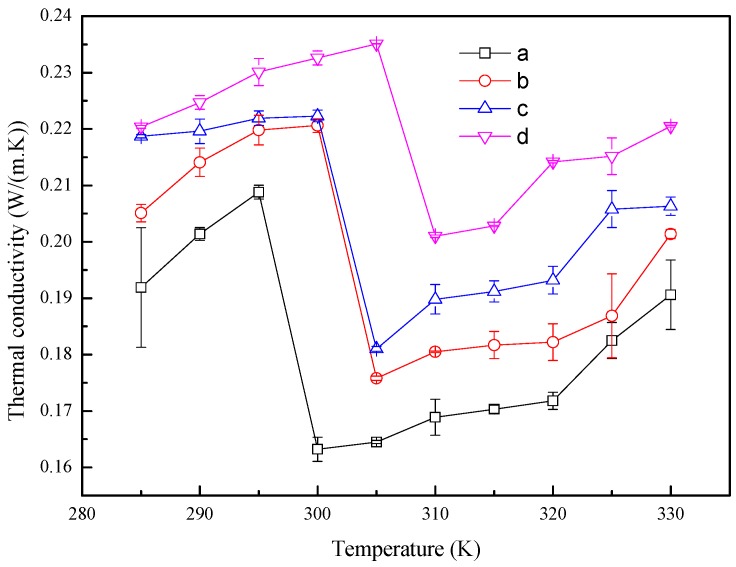
Thermal conductivities of the *n*-octadecane/Cu nanoparticle nanofluids with different temperatures. (**a**) 128 *n*-octadecane molecules; (**b**) 1 Cu nanoparticle and 512 *n*-octadecane molecules; (**c**) 1 Cu nanoparticle and 256 *n*-octadecane molecules; (**d**) 1 Cu nanoparticle and 128 *n*-octadecane molecules.

The thermal conductivities of the four systems are presented in Figure 10 which shows that the thermal conductivity increases with the temperature increase when the systems are in the solid or liquid state, while the thermal conductivity decreases sharply near the phase transition point. However, it indicates that the thermal conductivity of *n*-octadecane is enhanced by adding Cu nanoparticle into it and the thermal conductivity is large for high content of Cu nanoparticle. In the present study, the thermal conductivity of system (d) is about 14% larger than that of system (a) which consists of the basic fluid.

## 4. Conclusions

In this work, EMD is used to investigate the thermodynamic properties of *n*-octadecane/Cu nanoparticle nanofluids during phase transition, while NEMD is employed to study their thermal conductivity at the phase transition state. The results could draw the following conclusions.

The Cu nanoparticle could improve the thermal properties of *n*-octadecane. The diffusion coefficient at different temperatures indicates that the melting point of *n*-octadecane/Cu nanoparticle nanofluid increases with the increase of the mass ratio of the Cu nanoparticle. A similar trend for the heat capacity and thermal conductivity is also found. The reason is that the *n*-octadecane with Cu nanoparticles could store more energy than the pure *n*-octadecane system.

## Figures and Tables

**Table 1:** Thermal conductivity (W/(m·K)) of system (d) at 320 K.

NEMD	Maxwell
0.2142	0.2038
